# An exploratory, randomised, crossover study to investigate the effect of nicotine on cognitive function in healthy adult smokers who use an electronic cigarette after a period of smoking abstinence

**DOI:** 10.1186/s12954-024-00993-0

**Published:** 2024-04-06

**Authors:** Harry J. Green, Olivia K. O’Shea, Jack Cotter, Helen L. Philpott, Nik Newland

**Affiliations:** 1 Group Research and Development, British American Tobacco (Investments) Ltd, Regents Park Road, Southampton, SO15 8TL UK; 2 Simbec-Orion, Merthyr Tydfil, CF48 4DR UK

**Keywords:** Nicotine, E-cigarette, Cognitive function, Mood, Smoking cessation, Tobacco harm-reduction

## Abstract

**Background:**

As well as being associated with serious negative health outcomes, smoking has been reported to have an array of physiological and psychological effects, including effects on mood and cognitive function. Post-cessation, loss of such effects (including temporary deficits in cognitive function) have been cited as reasons for resumption of smoking. The effects of e-cigarettes and nicotine delivered by e-cigarettes on these functions have not been widely researched but may play a role in the effectiveness of e-cigarettes as a satisfactory alternative to combustible cigarettes for people who smoke, and in encouraging individuals who would otherwise continue to smoke, to transition to e-cigarettes.

**Methods:**

The study was an exploratory, randomised, partially-blinded, single-centre, five-arm crossover trial that recruited 40 healthy male and female people who smoke. At 5 study sessions, following a 12-h period of nicotine abstinence, participants were randomly assigned to use either a combustible cigarette, an e-cigarette of three varying nicotine strengths (18 mg/mL, 12 mg/mL or 0 mg/mL respectively) or observe a no product usage session. Participants completed pre- and post-product usage assessments to examine the product usage effect on cognitive performance (using the Cambridge Neuropsychological Test Automated Battery (CANTAB)), subjective mood and smoking urges.

**Results:**

A significant improvement in sustained attention task performance was observed following use of both the nicotine containing e-cigarettes and combustible cigarette compared to no product use. Additionally, there were no significant differences between the nicotine containing products, indicating that nicotine use enhanced sustained attention regardless of delivery format. Nicotine containing e-cigarette and combustible cigarette use also significantly improved overall mood of participants compared to no product use, with no significant differences observed between the nicotine containing products. Nicotine containing e-cigarette and combustible cigarette use significantly reduced smoking urges compared to no product use, though combustible cigarette use elicited the greatest reduction in smoking urges.

**Conclusions:**

Overall, the nicotine containing products improved sustained attention and mood while reducing smoking urges, with the studied e-cigarettes having comparable effects to combustible cigarettes across the assessed cognitive parameters and mood measures. These results demonstrate the potential role of e-cigarettes to provide an acceptable alternative for combustible cigarettes among people who would otherwise continue to smoke.

*Trial registration* ISRCTN (identifier: ISRCTN35376793).

**Supplementary Information:**

The online version contains supplementary material available at 10.1186/s12954-024-00993-0.

## Background

Whilst it is known that smoking is a leading avoidable cause of diseases including cardiovascular disease, lung disease and cancer, smoking prevalence in the majority of countries remains at 10–40% [1]. Despite the aforementioned health consequences being widely documented and smoking cessation medications having been available for several decades [2], smoking cessation rates remain generally low; in the US for example, based on data from 2018, successful rates were around 7.5% per year [3]. Seeking to lessen smoking’s impact on public health, a variety of regulatory and educational tobacco control initiatives have been utilised aiming to reduce global smoking rates [1]. To complement such approaches the policy of tobacco harm reduction (THR) has been adopted in certain markets. THR involves the switching of cigarette smoking with potentially reduced-risk nicotine products (RRPs) such as electronic cigarettes (e-cigarettes) [4]. Upon review of the available scientific evidence, both Public Health England and the UK Royal College of Physicians have stated that e-cigarette use is approximately 95% less harmful than cigarette smoking [5, 6] and regular review of the updated evidence has not changed this position [7]. Whilst THR has proved a successful strategy for many people who would have otherwise continued to smoke, there is a need to better understand the motivations of persons who continue to smoke and how loss of certain effects of nicotine post-cessation can inhibit their motivation to quit.

Smoking has been reported to have an array of physiological and psychological effects that some people who smoke identify as reasons for continuing to smoke, including effects on emotion and cognitive function [8, 9]. Nicotinic acetylcholine receptor activation via agonists, such as nicotine, can facilitate the release of an array of neurotransmitters that have been shown to be involved in cognitive functioning [10]. Functional brain imaging studies have demonstrated that nicotine is able to modulate frontal cortex activity (involved in higher-order cognitive functions such as sustained attention), the hippocampus (involved in episodic memory and working memory) and the amygdala (involved in emotional processes) [11]. A meta-analysis conducted assessing healthy adults (including persons who do and do not smoke) who were minimally tobacco-deprived, reported that nicotine may have significant positive effects on aspects of attention, motor abilities and memory [9].

Post-cessation of smoking and nicotine consumption, absence of such functional effects (including reported temporary deficits in cognitive function) [12] have been cited as reasons for resumption of smoking [13]. For example, difficulty concentrating, a component of nicotine withdrawal [14], has been reported as a factor that may lead to resumption of smoking [15]. Alterations to general mood and smoking urges have also been widely reported as reasons for resumption of smoking post-cessation [12, 13]. The effects of e-cigarettes (including delivery of nicotine) on these functions has not been widely researched but may play an important role in the acceptability of e-cigarettes for people who currently smoke in seeking alternatives to continued smoking, and may encourage individuals who would otherwise have continued to smoke, to switch completely to e-cigarettes instead.

This study aimed to compare the effect of smoking combustible cigarettes to that of e-cigarettes with varying levels of nicotine on cognitive functions (including sustained attention, episodic memory, working memory and executive function), general mood and cigarette smoking urges, in people who smoke after a period of nicotine abstinence. The hypothesis of the study was that cognitive performance and mood would be similar following use of the nicotine containing e-cigarettes and the combustible cigarette and differentiated from the impact of cessation and withdrawal of nicotine.

## Methods

A full description of the study protocol has been published previously [16]. The key aspects of the protocol are summarised below.

### Study design

The study was an exploratory, randomised, partially-blinded, single-centre, five-arm crossover trial. It was registered prospectively on ISRCTN (ISRCTN35376793) and received ethical approval from the *Wales Research Ethics Committee 1* (Cardiff, UK; reference 21/WA/0095). The research was performed in accordance with the Declaration of Helsinki (2013), Good Clinical Practice and applicable regulatory requirements. The trial was managed by Simbec-Orion and took place at their research facility located in Merthyr Tydfil, UK. Written informed consent was obtained from all participants prior to their inclusion in the study.

### Participants

At the screening visit, prospective participants were assessed for inclusion based on the trial eligibility criteria detailed in the protocol [16]. The main inclusion criteria included healthy male or female participant between 25 and 45 years of age; current smoker (self-reported consumption of at least 10 factory-made or self-rolled cigarettes per day (confirmed via urinary cotinine sample (> 200 ng/mL)) for 3 years or longer; familiar with e-cigarettes (classified as use for at least 1 month in the previous 2 years); BMI between 18.5 and 29.9 kg/m^2^ and considered in good general health (as confirmed via the principal investigator).

Main exclusion criteria included female participants who were pregnant or breastfeeding; participants who, prior to enrolment, were planning to quit or alter smoking/vaping usage within the duration of the study; participants with acute illness requiring treatment within 4 weeks prior to screening or upon admission; participants with a positive COVID-19 PCR (Antigen) test prior to Day 1; participants with evidence of renal, hepatic, central nervous system (CNS), respiratory, cardiovascular or metabolic dysfunction; participants who had been diagnosed with a clinically significant cognitive disorder (or had used a CNS enhancing or modulating medication in the prior 3 months) or participants with a colour vision deficiency (as determined by an Ishihara test performed at screening).

### Study products

The study products can be found in Table 1 (all study products were supplied by the trial sponsor). Participants received one of the study products at each of the 5 study visits. Participants used each of the study products or no product, once. A randomisation schedule was created (by Simbec-Orion) using a computer-generated pseudo-random permutation procedure in SAS version 9.4 to determine the order in which participants received the products; a randomisation code for 40 participants was produced based on a Williams Latin square design for a 5 × 5 crossover with 10 sequences, 4 participants per sequence.

**Table 1:** Overview of the study products

Product	Nicotine content	Product usage
No product usage	N/A	5-min rest period
E-cigarette (EPEN-0 mg)	0 mg/ml nicotine	5-min ad libitum
E-cigarette (EPEN-12 mg)	12 mg/ml nicotine	5-min ad libitum
E-cigarette (EPEN-18 mg)	18 mg/ml nicotine	5-min ad libitum
Combustible Cigarette	N/A*	1 stick 5-min ad libitum

^*^7 mg ISO tar cigarette

The e-cigarette study product (Vype ePen3; now branded as “Vuse ePen3”) is a commercially available, closed-system e-cigarette and was selected in part due to its known pharmacokinetic profile (the start of the cognitive testing was aligned with the product TMax) [17]. E-liquids used in the study contained 0, 12 or 18 mg/ml nicotine (protonated) respectively and were Golden Tobacco flavour; participants were blinded to the e-cigarette nicotine concentration (to conceal the specifics of the utilised study products, e-cigarette products were pre-assembled before being handed to participants). The cigarettes used in the study were commercially sourced Benson & Hedges Sky Blue king-size cigarettes, selected as they were the market leading combustible cigarette in the UK at the time of study set-up and their pharmacokinetic profile has been demonstrated previously [17].

### Outcome assessments

#### Cognitive assessments

The Cambridge Neuropsychological Test Automated Battery (CANTAB) (www.cambridgecognition.com) was used to assess participant’s cognitive function. These computerised tests have been developed and validated over the past 30 years and have demonstrated robust sensitivity to changes in cognitive performance in response to nutritional, pharmaceutical and digital interventions [18–22]. CANTAB tests have also been used to assess the impact of nicotine on cognitive function, both in healthy adult and clinical populations [23–25]. The CANTAB tests (the primary outcome measures) used in this trial are highlighted below and were delivered in the order shown:

- Rapid Visual Information Processing (RVP): assesses sustained attention (~ 7 min)
- Paired Associates Learning (PAL): assesses episodic memory (~ 10 min)
- Spatial Working Memory (SWM): assesses working memory (~ 7 min)
- One Touch Stockings of Cambridge (OTS): assesses executive function (specifically planning) (~ 12 min)

Drawing from published literature, the tests were selected based on their capacity to provide a broad assessment of the cognitive domains of interest assessing the relationship between nicotine and cognition [9] and their previously established sensitivity to the effects of nicotine administration and abstinence [23, 24, 32, 46]. The tests were administered using a touchscreen tablet computer (iPad 10.2 in., 32 GB, Apple Inc.) attached to a supporting stand. A detailed description of these tests and their associated outcome measures are provided in Additional file 1: File S1. All participants completed the full CANTAB test battery at a screening visit prior to their first testing session in order to familiarise them with the task demands and mitigate the impact of practice effects on cognitive task performance once the active study began. Furthermore, the CANTAB tests used in the current study contained randomized stimuli in order to ensure that participants completed parallel versions of the task at each assessment.

#### Participant self-reported measures

In addition to the cognitive function assessments, participants also completed the following self-reported assessments (secondary outcome measures):

The Subjective Emotion Questionnaire (*SEQ*) was used to assess mood. The *SEQ* includes a series of Visual Analogue Scale (VAS) questions, each consisting of a 100 mm line anchored at the beginning and end by opposing statements; the questionnaire was adapted based upon a previously published version [26]. As described in full in the Statistical Analysis Plan (SAP) [16], composite scores were generated, categorised as either “positive emotion scores” or “negative emotion scores”.

The Questionnaire of Smoking Urges-Brief (*QSU-B*) is a 10-item questionnaire widely used to assess smoking urges [27]. Composites are created that assess “Factor 1” (intention/desire to smoke) and “Factor 2” (relief of negative affect / urgent desire to smoke) scores respectively [27].

Due to potential confounding effects on cognitive outcomes, the effect of sleepiness and caffeine craving were also assessed before every study session using the Karolinska Sleepiness Scale (*KSS*) [28] and the Assessment of Caffeine Urges (*ACU*) questionnaire (adapted from [29]), respectively. The Fagerstrom Test for Nicotine Dependence (*FTND*) [30], the Tobacco Use History Questionnaire (*TUHQ*) (study specific questionnaire) and a Caffeine Consumption Questionnaire (*CCQ*) (adapted from [31]) were also completed prior to study session 1 only to determine each participant’s level of nicotine dependence, their typical tobacco use habits and their average caffeine consumption respectively. Finally, as product liking can influence mood outcomes, a 1 question product satisfaction questionnaire (*PSQ*) (study specific questionnaire) was administered after each product usage session.

### Study procedure overview

**Fig. 1:**
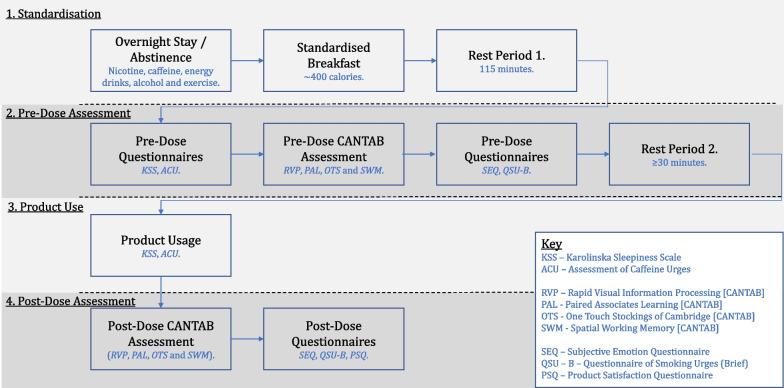
Study schematic overview. Study schematic highlighting the key steps of each study session. KSS, Karolinska sleepiness scale; ACU, Assessment of Caffeine Urges; RVP, Rapid Visual Information Processing; PAL, Paired Associates Learning; OTS, One Touch Stockings of Cambridge; SWM, Spatial Working Memory; SEQ, subjective emotion questionnaire; QSU-B, Questionnaire of Smoking Urges-brief; PSQ, product satisfaction questionnaire

A study design schematic is shown in Fig. 1 and the full overview has been published previously [16].

At each study visit participants were required to attend the research unit the day before the study session, staying at the unit overnight which ensured that participants abstained from any substance use (nicotine products, alcohol and caffeine). At study visit 1 (only), participants were familiarised with the full study testing procedures and asked to complete several baseline questionnaires (*FTND*, *CCQ* and the *TUHQ*).

On the morning of each study session, following a minimum of 12-h abstinence from smoking, nicotine products, alcohol and caffeine, participants were provided a standardised breakfast. Following a rest period, participants then completed two baseline questionnaires (*KSS* and the *ACU*). Participants then completed the pre-dose CANTAB assessment battery (*RVP*, *PAL*, *OTS* and *SWM*) immediately followed by completion of the subjective questionnaires (*SEQ* and *QSU-B*). Following a second rest period, participants self-administered one of the provided study products (or no product) in accordance with the randomisation schedule (e-cigarette devices were weighed immediately before and after use to calculate device mass loss (DML), a metric that correlates with the amount of vapour inhaled). At the end of the 5-min product (or no product) use period, participants repeated the CANTAB assessments. Participants then repeated the *SEQ* and *QSU-B*. Finally, a product satisfaction questionnaire was completed (this was not conducted following the no product use session).

A post-study follow-up telephone call was conducted with all participants 5–7 days after completion of their final study session.

### Statistical approach

The full SAP, including determination of the number of participants required to detect a statistically significant difference, has been published previously; the study enrolled 40 participants, allowing for attrition to ensure a minimum of 35 participants completed the study. All statistical analysis were performed using SAS version 9.4.

Statistical comparisons were performed between products using an analysis of variance (ANOVA) on both absolute and baseline-adjusted results. Correlation analyses (Pearson’s correlation coefficient) were used to examine associations between the cognitive and mood outcome measures with age, handedness, nicotine dependence, caffeine consumption / craving and sleepiness. Where the results of the correlation analysis suggested an association with a particular endpoint (correlation coefficient |r|≥ 0.5), the statistical analysis of the endpoint was repeated using an analysis of covariance (ANCOVA) including the parameter as a covariate (if utilised, this has been made clear in the footnotes of the below data tables). Unless otherwise stated, outcome measures are reported as baseline-adjusted mean differences between pre- and post- product usage.

## Results

### Participant demographics and nicotine consumption

The study randomised 40 healthy male and female people who smoke, with 37 (92.5%) completing all of the study visits (participant demographics are presented in Table 2). 3 participants did not complete all visits although data from the completed visits were retained in the analysis. Of these participants, 2 were removed for non-fulfilment of eligibility criteria (screened positive for drugs of abuse) and 1 requested early withdrawal. There were no major protocol deviations or significant compliance issues during the study. Additionally, there were no significant differences between groups at baseline on either the subjective measures (QSU-B, KSS, ACU, SEQ all *p* > 0.39) or CANTAB endpoints (all *p* > 0.27).

**Table 2:** Summary of participant demographic information

Parameter	Statistic	Overall (N = 40)
Age (yrs)	Mean	33.7
	SD	5.84
Gender		
Male	n (%)	23 (57.5)
Female	n (%)	17 (42.5)
Weight (kg)	Mean	77.09
	SD	13.316
BMI (kg/m^2^)	Mean	25.49
	SD	2.873
Race		
Caucasian	n (%)	40 (100.0)
Other	n (%)	0 (0.0)
Handedness		
Right	n (%)	34 (85.0)
Left	n (%)	6 (15.0)
FTND total Score	Mean	5.0
	SD	1.74
Cigarettes/day	Mean	13.4
	SD	3.43

The mean DML ranged from approximately 0.09–0.17 g for the three e-cigarette study products, with the greatest DML observed for the EPEN-0 mg. Estimated nicotine consumption was significantly higher following use of EPEN-18 mg compared with EPEN-12 mg (*p* = < 0.001) (Table 3).

**Table 3:** E-cigarette study product device mass loss and estimated nicotine consumption

Parameter	LSMean (95% CI)		
	EPEN-0 mg (n = 39)	EPEN-12 mg (n = 39)	EPEN-18 mg (n = 39)
Device mass loss (g)	0.166 (0.138, 0.194)	0.095 (0.075, 0.115)	0.085 (0.071, 0.099)
Estimated nicotine consumption (mg)	N/A	1.14 (0.896, 1.38)	1.53 (1.28, 1.77)

### CANTAB assessment scores

#### RVP

Table 4 shows a significantly greater increase in baseline-adjusted RVP A′ scores (a measure of signal detection) was observed following use of the EPEN-12 mg, EPEN-18 mg and combustible cigarette, compared to when no product was used, indicating an improvement in participants’ sustained attention following nicotine use. Similarly, a significantly greater increase in baseline-adjusted RVP A′ scores was observed following use of the EPEN-12 mg and combustible cigarette when compared to EPEN-0 mg (the comparison of EPEN-18 mg and EPEN-0 mg did not reach statistical significance (*p* = 0.0776)). However, there were no significant differences in the change in RVP A′ scores between the nicotine containing products (EPEN-12 mg, EPEN-18 mg and combustible cigarette) following product use (all *p* ≥ 0.96). Higher DML was significantly correlated with improved performance in the EPEN-12 mg condition (r = 0.43, *p* = 0.0064), but not the EPEN-18 mg condition (r = 0.00, *p* = 0.9998).

**Table 4:** RVP scores

Test product	Reference product	LSMean (95% CI)		Difference in LSMeans (95% CI) Test—Reference	Tukey adjusted *P*-value	Cohen’s d effect size
		Test product	Reference product			
EPEN-0 mg (n = 38)	No Product (n = 37)	0.00487 (− 0.00276, 0.01249)	0.00255 (− 0.00516, 0.01025)	0.00232 (− 0.01127, 0.01591)	0.9897	0.11
EPEN-12 mg (n = 39)	No Product (n = 37)	0.01950 (0.01199, 0.02702)	0.00255 (− 0.00516, 0.01025)	0.01696 (0.00345, 0.03046)	0.0061*	0.80
EPEN-18 mg (n = 39)	No Product (n = 37)	0.01752 (0.01000, 0.02504)	0.00255 (− 0.00516, 0.01025)	0.01497 (0.00146, 0.02848)	0.0218*	0.71
Combustible Cigarette (n = 39)	No Product (n = 37)	0.02068 (0.01317, 0.02820)	0.00255 (− 0.00516, 0.01025)	0.01814 (0.00464, 0.03164)	0.0027*	0.85
EPEN-12 mg (n = 39)	EPEN-0 mg (n = 38)	0.01950 (0.01199, 0.02702)	0.00487 (− 0.00276, 0.01249)	0.01464 (0.00120, 0.02807)	0.0253*	0.69
EPEN-18 mg (n = 39)	EPEN-0 mg (n = 38)	0.01752 (0.01000, 0.02504)	0.00487 (− 0.00276, 0.01249)	0.01265 (− 0.00084, 0.02614)	0.0776	0.60
Combustible Cigarette (n = 39)	EPEN-0 mg (n = 38)	0.02068 (0.01317, 0.02820)	0.00487 (− 0.00276, 0.01249)	0.01581 (0.00235, 0.02928)	0.0125*	0.74
EPEN-18 mg (n = 39)	EPEN-12 mg (n = 39)	0.01752 (0.01000, 0.02504)	0.01950 (0.01199, 0.02702)	− 0.00199 (− 0.01532, 0.01134)	0.9939	0.09
Combustible Cigarette (n = 39)	EPEN-12 mg (n = 39)	0.02068 (0.01317, 0.02820)	0.01950 (0.01199, 0.02702)	0.00118 (− 0.01215, 0.01451)	0.9992	0.06
Combustible Cigarette (n = 39)	EPEN-18 mg (n = 39)	0.02068 (0.01317, 0.02820)	0.01752 (0.01000, 0.02504)	0.00317 (− 0.01012, 0.01646)	0.9648	0.15

Results obtained using an ANOVA on post-product use results with fixed effects of product, period, sequence and a random effect of subject nested within sequence (*indicates *p* =  < 0.05). Participants with assessments flagged to indicate non-compliance, who also meet the performance outlier threshold (± 1.5 × IQR), have been excluded from the analysis of this data

ANOVA, analysis of variance; IQR, interquartile range

Baseline defined as − 85 to − 45 min pre-product use

#### SWM, OTS and PAL

There were no other significant differences in performance between conditions across any of the other baseline-adjusted CANTAB outcome measures included in the analyses (see Additional file 1: File S2).

#### Global composite scores

A significant increase in baseline-adjusted global accuracy (composite score) was observed following use of the combustible cigarette compared to when no product was used (*p* = 0.003) and when compared with EPEN-0 mg (*p* = 0.0489). There were no significant differences in global accuracy scores following use of either of the nicotine containing EPEN’s compared to when participants had used the combustible cigarette (all *p* ≥ 0.47).

No significant differences in global speed composite scores were observed between conditions.

### Participant self-reported measures

#### SEQ

As displayed in Table 5, both a significant increase in baseline-adjusted positive emotion and a significant reduction in baseline-adjusted negative emotion scores were observed following use of the EPEN-12 mg, EPEN-18 mg and combustible cigarette, compared to when no product was used. This indicates an improvement in participant-reported mood following nicotine use. Additionally, there were no significant differences in the change in positive emotion (all *p* ≥ 0.62) or negative emotion scores (all *p* ≥ 0.79) following use of either of the nicotine containing EPEN’s compared to when participants had used the combustible cigarette. The data show a consistent trend where higher nicotine levels in the e-cigarette product are associated with improvements in the calculated point estimates for both positive and negative emotion scores, although the individual differences did not reach statistical significance following use of any of the nicotine-containing products when compared to the nicotine-free e-cigarette (EPEN-0 mg).

**Table 5:** SEQ Scores

	Test product	Reference product	LSMean (95% CI)		Difference in LSMeans (95% CI) Test—Reference	Tukey Adjusted *P*-value	Cohen’s d effect size
			Test product	Reference product			
Positive Emotion Score	EPEN-0 mg (n = 38)	No Product (n = 37)	5.4 (0.7, 10.2)	− 0.9 (− 5.8, 4.0)	6.4 (− 1.5, 14.2)	0.1743	0.52
	EPEN-12 mg (n = 39)	No Product (n = 37)	8.0 (3.3, 12.8)	− 0.9 (− 5.8, 4.0)	8.9 (1.0, 16.9)	0.0190*	0.73
	EPEN-18 mg (n = 39)	No Product (n = 37)	11.1 (6.4, 15.8)	− 0.9 (− 5.8, 4.0)	12.0 (4.1, 19.9)	0.0004*	0.98
	Combustible Cigarette (n = 39)	No Product (n = 37)	12.0 (7.2, 16.7)	− 0.9 (− 5.8, 4.0)	12.9 (4.9, 20.8)	0.0001*	1.05
	EPEN-12 mg (n = 39)	EPEN-0 mg (n = 38)	8.0 (3.3, 12.8)	5.4 (0.7, 10.2)	2.6 (− 5.2, 10.4)	0.8919	0.21
	EPEN-18 mg (n = 39)	EPEN-0 mg (n = 38)	11.1 (6.4, 15.8)	5.4 (0.7, 10.2)	5.7 (− 2.1, 13.5)	0.2693	0.46
	Combustible Cigarette (n = 39)	EPEN-0 mg (n = 38)	12.0 (7.2, 16.7)	5.4 (0.7, 10.2)	6.5 (− 1.3, 14.3)	0.1506	0.53
	EPEN-18 mg (n = 39)	EPEN-12 mg (n = 39)	11.1 (6.4, 15.8)	8.0 (3.3, 12.8)	3.1 (− 4.6, 10.8)	0.8029	0.25
	Combustible Cigarette (n = 39)	EPEN-12 mg (n = 39)	12.0 (7.2, 16.7)	8.0 (3.3, 12.8)	3.9 (− 3.8, 11.7)	0.6222	0.32
	Combustible Cigarette (n = 39)	EPEN-18 mg (n = 39)	12.0 (7.2, 16.7)	11.1 (6.4, 15.8)	0.8 (− 6.8, 8.5)	0.9981	0.07
Negative Emotion Score	EPEN-0 mg (n = 38)	No Product (n = 37)	− 6.3 (− 11.0, − 1.7)	− 1.4 (− 6.2, 3.4)	− 5.0 (− 12.7, 2.8)	0.3997	0.41
	EPEN-12 mg (n = 39)	No Product (n = 37)	− 10.0 (− 14.7, − 5.4)	− 1.4 (− 6.2, 3.4)	− 8.7 (− 16.5, − 0.8)	0.0220*	0.71
	EPEN-18 mg (n = 39)	No Product (n = 37)	− 11.8 (− 16.4, − 7.2)	− 1.4 (− 6.2, 3.4)	− 10.4 (− 18.2, − 2.6)	0.0028*	0.86
	Combustible Cigarette (n = 39)	No Product (n = 37)	− 13.1 (− 17.8, − 8.5)	− 1.4 (− 6.2, 3.4)	− 11.8 (− 19.6, − 3.9)	0.0005*	0.97
	EPEN-12 mg (n = 39)	EPEN-0 mg (n = 38)	− 10.0 (− 14.7, − 5.4)	− 6.3 (− 11.0, − 1.7)	− 3.7 (− 11.4, 4.0)	0.6714	0.31
	EPEN-18 mg (n = 39)	EPEN-0 mg (n = 38)	− 11.8 (− 16.4, − 7.2)	− 6.3 (− 11.0, − 1.7)	− 5.5 (− 13.1, 2.2)	0.2891	0.45
	Combustible Cigarette (n = 39)	EPEN-0 mg (n = 38)	− 13.1 (− 17.8, − 8.5)	− 6.3 (− 11.0, − 1.7)	− 6.8 (− 14.5, 0.9)	0.1109	0.56
	EPEN-18 mg (n = 39)	EPEN-12 mg (n = 39)	− 11.8 (− 16.4, − 7.2)	− 10.0 (− 14.7, − 5.4)	− 1.8 (− 9.4, 5.9)	0.9687	0.15
	Combustible Cigarette (n = 39)	EPEN-12 mg (n = 39)	− 13.1 (− 17.8, − 8.5)	− 10.0 (− 14.7, − 5.4)	− 3.1 (− 10.7, 4.5)	0.7936	0.26
	Combustible Cigarette (n = 39)	EPEN-18 mg (n = 39)	− 13.1 (− 17.8, − 8.5)	− 11.8 (− 16.4, − 7.2)	− 1.3 (− 8.9, 6.3)	0.9884	0.11

Results obtained using an ANCOVA with fixed effects of product, period, sequence and a random effect of subject nested within sequence with the baseline value as a covariate (*indicates *p* =  < 0.05). Baseline defined as Pre-IP Use.

#### Smoking urges questionnaire

A full description of the smoking urges data is provided in Additional file 1: File S3.

In summary, a significant reduction in baseline-adjusted Factor 1 (intention/desire to smoke) and Factor 2 (relief of negative affect/urgent desire to smoke) scores was observed following EPEN-12 mg, EPEN-18 mg and combustible cigarette use when compared to when no product was used (all *p* < 0.0001), indicating a participant perceived reduction of smoking urges following nicotine use (comparison of EPEN-0 mg and no product use did not reach significance for either factor (*p* = 0.1186 and *p* = 0.0568, respectively)). When compared with EPEN-0 mg, EPEN-18 mg and combustible cigarette use induced a significant reduction in Factor 1 and Factor 2 scores respectively (all *p* ≤ 0.0011). Comparison of EPEN-12 mg and EPEN-0 mg use did not reach significance for either factor (*p* = 0.0602 and *p* = 0.1671, respectively). Combustible cigarette use induced a significantly greater reduction in Factor 1 scores compared with EPEN-12 mg (*p* < 0.0001) and EPEN-18 mg (*p* = 0.0294) and a significant reduction in Factor 2 scores compared to the EPEN-12 mg only (*p* = 0.0133).

### Adverse events

There were a total of 28 treatment-emergent adverse events reported by 21 (52.5%) of the participants across the duration of the study; the most common of which was headache (20%). The frequency of these adverse events was comparable across the conditions and considered unrelated to study product administration. No serious adverse events were reported.

## Discussion

The purpose of this trial was to compare the effect of nicotine containing e-cigarettes on cognitive functions, general mood and smoking urges to the effect elicited by a combustible cigarette (in people who smoke) after a period of nicotine abstinence. Overall, the nicotine containing e-cigarettes had comparable effects to a combustible cigarette across the assessed cognitive parameters and mood measures. This discovery is significant, highlighting the potential of such alternatives to serve as a suitable replacement for combustible cigarettes, encouraging individuals who currently smoke and would otherwise continue to do so, to transition to e-cigarettes.

A significant improvement in RVP A’ performance was observed following use of all nicotine containing products when compared to no product, indicating enhanced sustained attention following nicotine use. Review of the associated effect sizes indicated that the magnitude of these differences were all in the medium-to-large range (see Table 4) (Cohen’s d = 0.71–0.85). This finding is consistent with previous research. For example, both Austin [23] and Jackson [24] demonstrated enhanced performance on the CANTAB RVP task in satiated (compared with abstinent) people who smoke. Similarly, research has shown that use of very low nicotine cigarettes negatively affected sustained attention on this task when compared with usual brand cigarettes [32]. Nicotine administered in other formats (e.g. gum and nasal spray) has also been shown to improve performance on attentional tasks [33–35]. These results are also consistent with neuroimaging studies which suggest that nicotine administration can modulate the networks underlying attentional processes [11].

No significant changes were observed on the other cognitive outcomes. This is reflective of the broader nicotine literature which shows significant heterogeneity between studies with regards to the impact of nicotine on other aspects of cognitive function. For example, while several studies have reported that people who smoke may experience benefits to their working memory following nicotine use [36, 37], others have failed to find this effect [38, 39] or even observed the opposite [40]. It is unclear why, in the current study, these aspects of cognition were not impacted by nicotine use (or seemingly withdrawal) and further research is required to establish the reason. The absence of changes in the other CANTAB assessments suggests it may be specifically the ability to sustain engagement in this repetitive attentional task that was enhanced, as opposed to the briefer engagement required in the other, arguably more varied and engaging CANTAB assessments.

A significant improvement was also reported in general mood measures following use of each of the nicotine containing products, with no significant differences observed between these products (although there was a trend towards improvement with increasing nicotine level). Again, this finding is consistent with previously published literature that has demonstrated that nicotine, delivered in various formats, can enhance mood and reduce perceived negative affect in people who smoke following a period of nicotine abstinence [35, 41, 42]. In regard to smoking urges, nicotine products reduced smoking urges in a seemingly dose dependent manner; for Factor 1 scores, the cigarette performed significantly better than the nicotine containing e-cigarettes, which, in turn, also performed significantly better than no product. These findings align with previously published research which has demonstrated the ability of nicotine containing e-cigarettes to reduce smoking urges [36, 42]. A recent Cochrane review has also concluded that there is high-certainty evidence that e-cigarettes increase quit rates compared to traditional nicotine replacement therapies (NRTs) [43], indicating the potential importance of the nicotine delivery format.

There was a lack of significant differences in mood changes between the nicotine containing products and the nicotine-free e-cigarette (though these trended numerically in the anticipated direction). This was despite the former demonstrating robust improvements in both positive and negative mood relative to the ‘no product’ condition. This suggests that the act of vaping an e-cigarette may exert some form of placebo effect on mood among abstinent people who smoke. Given that perceived cognitive difficulties are often cited as a barrier to quitting [13], and it has been reported that mood may be a stronger correlate of perceived cognitive performance than objective performance [44, 45], it also raises the question as to what extent these cognitive improvements and decrements may be overestimated by people who smoke. Consistent with this, studies have suggested that the magnitude of the objective pro-cognitive benefits that are experienced by people who smoke following nicotine use are typically in the ‘small’ effect-size range [9]. Alternatively, it may be that beneficial mood effects, in part, contribute to enhanced objective cognitive task performance, for example, by enhancing motivation coupled with reduced distraction due to cravings. This, in part, may help to explain why the improvement in RVP task performance in the EPEN-18 mg condition met only trend-level significance when compared to the change in performance observed within the EPEN-0 mg condition.

This study is among the first randomised trials to compare the effects of different nicotine strength e-cigarettes (including a nicotine-free placebo) with combustible cigarettes on cognitive, mood measures or smoking urges in people who smoke following a period of abstinence. Overall, the results were broadly consistent with two similar studies regarding reduction in smoking urges and improvement in mood following nicotine usage, although there were some discrepancies in the observed cognitive outcomes [36, 38]. CANTAB tasks were selected as the cognitive assessments for the current trial based on their established sensitivity to the effects of nicotine abstinence and administration [23, 24, 32, 46], as well as their extensive use in clinical trials [21, 22, 47], including acute-dose studies in healthy adults [19, 20, 48]. A possible limitation of the study is the varying quantities of nicotine consumed between participants within the conditions (due to ad libitum use). However, *ad lib* usage was selected as it replicates normal behaviour and real-world participant usage. Secondly, the order of the CANTAB tasks was designed to align the task most likely to be sensitive to nicotine administration (RVP) with the product TMax. As such, a possible consideration for the absence of effects on other measures may be partially explained by the fact that later tasks will have been initiated some time (~ 8–25 min) after nicotine consumption and hence, nicotine levels may have been lower at this time [17]. Finally, the inability to blind both the cigarette and no product arms is a limitation worth noting. However, the inclusion of the nicotine-free e-cigarette offered a unique approach to explore the potential impact of placebo effects in this context.

## Conclusions

Overall, this study has demonstrated that, in dependent people who smoke but whom were abstinent from smoking and nicotine consumption, nicotine containing e-cigarettes and combustible cigarettes were able to improve sustained attention, mood and reduce smoking urges, with e-cigarettes having a comparable effect to combustible cigarettes across the assessed cognitive parameters and mood measures. Whilst the studied e-cigarettes are neither licensed nor marketed as smoking cessation devices, this research is important as the reported effects of the e-cigarettes may demonstrate the acceptability of e-cigarettes as a satisfactory alternative to continued smoking for some people who smoke.

### Supplementary Information

**Additional file 1**. Summary of the Cambridge Neuropsychological Test Automated Battery (CANTAB) – Task and key outcome measure descriptions.

## Data Availability

The datasets analysed during the current study are available from the corresponding author on reasonable request.

## Abbreviations

CANTAB: Cambridge Neuropsychological Test Automated Battery
E-cigarette: Electronic cigarette
THR: Tobacco harm reduction
RRP’s: Reduced-risk nicotine products
CNS: Central nervous system
TMax: Time of Maximum Plasma Nicotine Concentration
RVP: Rapid Visual Information Processing
PAL: Paired Associates Learning
SWM: Spatial Working Memory
OTS: One Touch Stockings of Cambridge
SEQ: Subjective emotion questionnaire
VAS: Visual analogue scale
QSU-B: Questionnaire of Smoking Urges-brief
KSS: Karolinska sleepiness scale
ACU: Assessment of Caffeine Urges
FTND: The Fagerstrom test for nicotine dependence
TUHQ: Tobacco Use History questionnaire
CCQ: Caffeine Consumption questionnaire
PSQ: Product satisfaction questionnaire
DML: Device mass loss
SAP: Statistical analysis plan
ANOVA: Analysis of variance
ANCOVA: Analysis of covariance
RVP A′: RVP A′ Prime
IQR: Interquartile range
NRTs: Nicotine replacement therapies
