# Supplementary material for: An exploratory, randomised, crossover study to investigate the effect of nicotine on cognitive function in healthy adult smokers who use an electronic cigarette after a period of smoking abstinence
Source: Harm Reduct J. 2024 Apr 6;21:78. doi: 10.1186/s12954-024-00993-0 (PMC10998423; doi:10.1186/s12954-024-00993-0)
Supplement: Supplementary file 1 — Additional file 1. Summary of the Cambridge Neuropsychological Test Automated Battery (CANTAB) – Task and key outcome measure descriptions. [file 12954_2024_993_MOESM1_ESM.docx]

***File S1***

*The Cambridge Neuropsychological Test Automated Battery (CANTAB) – Task and key outcome measure descriptions*

1. Rapid Visual Information Processing (RVP)

RVP is a sensitive measure of sustained attention, outputting measures of response accuracy, target sensitivity and reaction times. During the task, single digits are presented in a pseudo-random order (100 digits per minute) and participants must detect a series of 3-digit sequences (e.g. 3-5-7; 2-4-6; 4-6-8), responding when they see the final number of the sequence. Nine target sequences are presented every minute.

*Key outcome measures:*

RVPA – RVP A’ Prime: The signal detection measure of a participant’s sensitivity to the target sequence (string of three numbers), regardless of response tendency (the expected range is 0.00 to 1.00; low to high performance).

RVPFFA – RVP Probability of False Alarm: The number of sequence presentations that were false alarms divided by the number of sequence presentations that were false alarms plus the number of sequence presentations that were correct rejections (False Alarms ÷ (False Alarms + Correct Rejections)).

RVPMDL – RVP Median Latency: The median response latency on trials where the participant responded correctly. Calculated across all assessed trials.

1. Paired Associates Learning (PAL)

PAL is a measure of episodic memory. During the task, boxes are displayed on the screen and open in turn to reveal a pattern. Once all of the boxes have been opened, each pattern is shown in the centre of the screen in a randomised order. Participants are instructed to select the box in which the pattern on the screen was initially located. If an error is made, the patterns in each box are re-presented to participants. Once the participant has completed the stage, the test progresses in difficulty presenting an increasing number of patterns. For participants who fail to complete all levels, an adjusted total is calculated that allows for errors predicted in the stages that were not attempted.

*Key outcome measures:*

PALTEA – PAL Total Attempts Adjusted: The number of times the participant chose the incorrect box for a stimulus on assessment problems (PALTE), plus an adjustment for the estimated number of errors they would have made on any problems, attempts and recalls they did not reach. This measure allows comparison of performance on errors made across all participants regardless of those who terminated early versus those completing the final stage of the task. The more errors made, the poorer the performance.

PALFAMS – PAL First Attempt Memory Score: The number of times a participant chose the correct box on their first attempt when recalling the pattern locations. Calculated across all assessed trials.

1. Spatial Working Memory (SWM)

SWM assesses a participant’s ability to retain spatial information and manipulate it in working memory. During the task, a number of coloured boxes are presented on the screen and the software “hides” a token in these boxes one at a time. The participant is instructed to select the boxes in turn to search for a hidden token. When a token is found it is placed in a storage area on the right-hand side of the screen. The participant then searches for more tokens until the same number of tokens are found as the number of boxes. The software will never hide a token in the same box twice within a given task. As the test progresses, it increases in difficulty, presenting an increasing number of boxes.

*Key outcome measures:*

SWMBE – SWM Between Errors: The number of times the participant incorrectly revisits a box in which a token has previously been found. Calculated across all assessed four, six and eight token trials. The more errors, the poorer the performance.

SWMS – SWM Strategy: The number of times a participant begins a new search pattern from the same box they started with previously. If they always begin a search from the same starting point, it is inferred that the subject is employing a planned strategy for finding the tokens. Therefore, a low score indicates high strategy use, a high score indicates that they are beginning their searches from many different boxes. Calculated across assessed trials with 6 tokens or more.

1. One Touch Stockings of Cambridge (OTS)

OTS is a test of executive function. During the task, participants see two displays containing three coloured balls. The displays are presented in such a way that they can easily be perceived as stacks of coloured balls held in stockings suspended from a beam. Below this is presented a row of numbered boxes. An animated tutorial first demonstrates to the participant how to use the balls in the lower display to copy the pattern in the upper display, and completes one demonstration problem, where the solution requires two moves. The participant must then complete two further problems, one each of three moves and four moves. Next the participant is shown further problems and must work out the minimum number of moves required to solve the problems, selecting the appropriate box at the bottom of the screen to indicate their response.

*Key outcome measures:*

OTSPSFC – OTS Problems Solved on First Choice: The total number of assessed trials where the subject chose the correct answer on their first attempt. Calculated across all assessed trials.

OTSMDLFC – OTS Median Latency to First Choice: The median latency, measured from the appearance of the stocking balls until the first box choice was made by the subject. Calculated across all assessed trials where the subject's first response was correct.

***File S2***

*Summary of the Statistical Analysis of Baseline-Adjusted CANTAB Outcome Measures (SWM, OTS & PAL).*

1. RVP
   1. *RVP Probability of False Alarm (RVPFFA)*

| **Test Product** | **Reference Product** | **LSMean (95% CI)** | | **Difference in LSMeans (95% CI) Test ‑ Reference** | **Tukey Adjusted P‑value** |
| --- | --- | --- | --- | --- | --- |
|  |  | **Test Product** | **Reference Product** |  |  |
| EPEN-0mg (n=38) | No Product (n=37) | 0.00012 (‑0.00183, 0.00207) | 0.00143 (‑0.00054, 0.00340) | ‑0.00131 (‑0.00370, 0.00107) | 0.5508 |
| EPEN-12mg (n=39) | No Product (n=37) | ‑0.00080 (‑0.00274, 0.00113) | 0.00143 (‑0.00054, 0.00340) | ‑0.00224 (‑0.00460, 0.00013) | 0.0741 |
| EPEN-18mg (n=39) | No Product (n=37) | ‑0.00017 (‑0.00211, 0.00177) | 0.00143 (‑0.00054, 0.00340) | ‑0.00160 (‑0.00399, 0.00079) | 0.3470 |
| B734 (n=39) | No Product (n=37) | ‑0.00044 (‑0.00238, 0.00150) | 0.00143 (‑0.00054, 0.00340) | ‑0.00187 (‑0.00425, 0.00050) | 0.1930 |
| EPEN-12mg (n=39) | EPEN-0mg (n=38) | ‑0.00080 (‑0.00274, 0.00113) | 0.00012 (‑0.00183, 0.00207) | ‑0.00092 (‑0.00326, 0.00142) | 0.8108 |
| EPEN-18mg (n=39) | EPEN-0mg (n=38) | ‑0.00017 (‑0.00211, 0.00177) | 0.00012 (‑0.00183, 0.00207) | ‑0.00029 (‑0.00265, 0.00206) | 0.9970 |
| B734 (n=39) | EPEN-0mg (n=38) | ‑0.00044 (‑0.00238, 0.00150) | 0.00012 (‑0.00183, 0.00207) | ‑0.00056 (‑0.00291, 0.00179) | 0.9642 |
| EPEN-18mg (n=39) | EPEN-12mg (n=39) | ‑0.00017 (‑0.00211, 0.00177) | ‑0.00080 (‑0.00274, 0.00113) | 0.00063 (‑0.00171, 0.00297) | 0.9450 |
| B734 (n=39) | EPEN-12mg (n=39) | ‑0.00044 (‑0.00238, 0.00150) | ‑0.00080 (‑0.00274, 0.00113) | 0.00036 (‑0.00197, 0.00269) | 0.9929 |
| B734 (n=39) | EPEN-18mg (n=39) | ‑0.00044 (‑0.00238, 0.00150) | ‑0.00017 (‑0.00211, 0.00177) | ‑0.00027 (‑0.00259, 0.00205) | 0.9976 |

Results obtained using an ANOVA on post‑product use results with fixed effects of product, period, sequence and a random effect of subject nested within sequence. Subjects with assessments flagged to indicate non‑compliance, who also meet the performance outlier threshold (+/‑ 1.5 x IQR), have been excluded from the analysis of this data

ANOVA = analysis of variance, IQR = interquartile range. Baseline defined as ‑85 to ‑45 min pre‑product use.

- 1. *RVP Median Latency (RVPMDL)*

| **Test Product** | **Reference Product** | **LSMean (95% CI)** | | **Difference in LSMeans (95% CI) Test ‑ Reference** | **Tukey Adjusted P‑value** |
| --- | --- | --- | --- | --- | --- |
|  |  | **Test Product** | **Reference Product** |  |  |
| EPEN-0mg (n=38) | No Product (n=37) | ‑15.65518 (‑28.35143, ‑2.95893) | ‑17.48371 (‑30.28125, ‑4.68616) | 1.82853 (‑15.23031, 18.88736) | 0.9983 |
| EPEN-12mg (n=39) | No Product (n=37) | ‑16.69596 (‑29.28579, ‑4.10613) | ‑17.48371 (‑30.28125, ‑4.68616) | 0.78774 (‑16.19426, 17.76975) | 0.9999 |
| EPEN-18mg (n=39) | No Product (n=37) | ‑19.76492 (‑32.36082, ‑7.16902) | ‑17.48371 (‑30.28125, ‑4.68616) | ‑2.28122 (‑19.26303, 14.70060) | 0.9959 |
| B734 (n=39) | No Product (n=37) | ‑27.20468 (‑39.79793, ‑14.61143) | ‑17.48371 (‑30.28125, ‑4.68616) | ‑9.72097 (‑26.72037, 7.27842) | 0.5127 |
| EPEN-12mg (n=39) | EPEN-0mg (n=38) | ‑16.69596 (‑29.28579, ‑4.10613) | ‑15.65518 (‑28.35143, ‑2.95893) | ‑1.04078 (‑17.87488, 15.79331) | 0.9998 |
| EPEN-18mg (n=39) | EPEN-0mg (n=38) | ‑19.76492 (‑32.36082, ‑7.16902) | ‑15.65518 (‑28.35143, ‑2.95893) | ‑4.10974 (‑21.01625, 12.79677) | 0.9622 |
| B734 (n=39) | EPEN-0mg (n=38) | ‑27.20468 (‑39.79793, ‑14.61143) | ‑15.65518 (‑28.35143, ‑2.95893) | ‑11.54950 (‑28.49393, 5.39493) | 0.3311 |
| EPEN-18mg (n=39) | EPEN-12mg (n=39) | ‑19.76492 (‑32.36082, ‑7.16902) | ‑16.69596 (‑29.28579, ‑4.10613) | ‑3.06896 (‑19.83983, 13.70191) | 0.9867 |
| B734 (n=39) | EPEN-12mg (n=39) | ‑27.20468 (‑39.79793, ‑14.61143) | ‑16.69596 (‑29.28579, ‑4.10613) | ‑10.50872 (‑27.28802, 6.27058) | 0.4188 |
| B734 (n=39) | EPEN-18mg (n=39) | ‑27.20468 (‑39.79793, ‑14.61143) | ‑19.76492 (‑32.36082, ‑7.16902) | ‑7.43976 (‑24.15406, 9.27454) | 0.7340 |

Results obtained using an ANOVA on post‑product use results with fixed effects of product, period, sequence and a random effect of subject nested within sequence. Subjects with assessments flagged to indicate non‑compliance, who also meet the performance outlier threshold (+/‑ 1.5 x IQR), have been excluded from the analysis of this data

ANOVA = analysis of variance, IQR = interquartile range. Baseline defined as ‑85 to ‑45 min pre‑product use.

1. SWM
   1. *SWM Between Errors (SWMBE)*

| **Test Product** | **Reference Product** | **LSMean (95% CI)** | | **Difference in LSMeans (95% CI) Test ‑ Reference** | **Tukey Adjusted P‑value** |
| --- | --- | --- | --- | --- | --- |
|  |  | **Test Product** | **Reference Product** |  |  |
| EPEN-0mg (n=38) | No Product (n=37) | 1.0 (‑0.8, 2.7) | 0.1 (‑1.7, 1.9) | 0.9 (‑1.9, 3.7) | 0.9120 |
| EPEN-12mg (n=39) | No Product (n=37) | 0.2 (‑1.5, 2.0) | 0.1 (‑1.7, 1.9) | 0.1 (‑2.7, 2.9) | 0.9999 |
| EPEN-18mg (n=39) | No Product (n=37) | ‑0.8 (‑2.6, 1.0) | 0.1 (‑1.7, 1.9) | ‑0.9 (‑3.7, 2.0) | 0.9175 |
| B734 (n=39) | No Product (n=37) | 0.5 (‑1.3, 2.3) | 0.1 (‑1.7, 1.9) | 0.4 (‑2.4, 3.2) | 0.9928 |
| EPEN-12mg (n=39) | EPEN-0mg (n=38) | 0.2 (‑1.5, 2.0) | 1.0 (‑0.8, 2.7) | ‑0.7 (‑3.5, 2.1) | 0.9503 |
| EPEN-18mg (n=39) | EPEN-0mg (n=38) | ‑0.8 (‑2.6, 1.0) | 1.0 (‑0.8, 2.7) | ‑1.7 (‑4.5, 1.1) | 0.4242 |
| B734 (n=39) | EPEN-0mg (n=38) | 0.5 (‑1.3, 2.3) | 1.0 (‑0.8, 2.7) | ‑0.4 (‑3.2, 2.3) | 0.9924 |
| EPEN-18mg (n=39) | EPEN-12mg (n=39) | ‑0.8 (‑2.6, 1.0) | 0.2 (‑1.5, 2.0) | ‑1.0 (‑3.8, 1.8) | 0.8557 |
| B734 (n=39) | EPEN-12mg (n=39) | 0.5 (‑1.3, 2.3) | 0.2 (‑1.5, 2.0) | 0.3 (‑2.5, 3.1) | 0.9984 |
| B734 (n=39) | EPEN-18mg (n=39) | 0.5 (‑1.3, 2.3) | ‑0.8 (‑2.6, 1.0) | 1.3 (‑1.5, 4.1) | 0.6934 |

Results obtained using an ANOVA on post‑product use results with fixed effects of product, period, sequence and a random effect of subject nested within sequence. Subjects with assessments flagged to indicate non‑compliance, who also meet the performance outlier threshold (+/‑ 1.5 x IQR), have been excluded from the analysis of this data.

(ANOVA = analysis of variance, IQR = interquartile range). Baseline defined as ‑85 to ‑45 min pre‑product use.

- 1. *SWM Strategy (SWMS)*

| **Test Product** | **Reference Product** | **LSMean (95% CI)** | | **Difference in LSMeans (95% CI) Test ‑ Reference** | **Tukey Adjusted P‑value** |
| --- | --- | --- | --- | --- | --- |
|  |  | **Test Product** | **Reference Product** |  |  |
| EPEN-0mg (n=38) | No Product (n=37) | ‑0.2 (‑0.7, 0.4) | ‑0.4 (‑1.0, 0.1) | 0.2 (‑0.8, 1.3) | 0.9719 |
| EPEN-12mg (n=39) | No Product (n=37) | ‑0.1 (‑0.6, 0.5) | ‑0.4 (‑1.0, 0.1) | 0.4 (‑0.7, 1.4) | 0.8687 |
| EPEN-18mg (n=39) | No Product (n=37) | ‑0.6 (‑1.1, 0.0) | ‑0.4 (‑1.0, 0.1) | ‑0.2 (‑1.2, 0.9) | 0.9924 |
| B734 (n=39) | No Product (n=37) | 0.1 (‑0.5, 0.6) | ‑0.4 (‑1.0, 0.1) | 0.5 (‑0.6, 1.5) | 0.7090 |
| EPEN-12mg (n=39) | EPEN-0mg (n=38) | ‑0.1 (‑0.6, 0.5) | ‑0.2 (‑0.7, 0.4) | 0.1 (‑0.9, 1.2) | 0.9968 |
| EPEN-18mg (n=39) | EPEN-0mg (n=38) | ‑0.6 (‑1.1, 0.0) | ‑0.2 (‑0.7, 0.4) | ‑0.4 (‑1.4, 0.6) | 0.8243 |
| B734 (n=39) | EPEN-0mg (n=38) | 0.1 (‑0.5, 0.6) | ‑0.2 (‑0.7, 0.4) | 0.2 (‑0.8, 1.3) | 0.9661 |
| EPEN-18mg (n=39) | EPEN-12mg (n=39) | ‑0.6 (‑1.1, 0.0) | ‑0.1 (‑0.6, 0.5) | ‑0.5 (‑1.6, 0.5) | 0.6134 |
| B734 (n=39) | EPEN-12mg (n=39) | 0.1 (‑0.5, 0.6) | ‑0.1 (‑0.6, 0.5) | 0.1 (‑0.9, 1.1) | 0.9981 |
| B734 (n=39) | EPEN-18mg (n=39) | 0.1 (‑0.5, 0.6) | ‑0.6 (‑1.1, 0.0) | 0.6 (‑0.4, 1.7) | 0.4168 |

Results obtained using an ANOVA on post‑product use results with fixed effects of product, period, sequence and a random effect of subject nested within sequence. Subjects with assessments flagged to indicate non‑compliance, who also meet the performance outlier threshold (+/‑ 1.5 x IQR), have been excluded from the analysis of this data

(ANOVA = analysis of variance, IQR = interquartile range). Baseline defined as ‑85 to ‑45 min pre‑product use.

1. OTS
   1. *OTS Problems Solved on First Choice (OTSPSFC)*

| **Test Product** | **Reference Product** | **LSMean (95% CI)** | | **Difference in LSMeans (95% CI) Test ‑ Reference** | **Tukey Adjusted P‑value** |
| --- | --- | --- | --- | --- | --- |
|  |  | **Test Product** | **Reference Product** |  |  |
| EPEN-0mg (n=37) | No Product (n=37) | ‑0.2 (‑1.1, 0.6) | ‑0.4 (‑1.3, 0.4) | 0.2 (‑0.8, 1.2) | 0.9779 |
| EPEN-12mg (n=37) | No Product (n=37) | ‑0.4 (‑1.2, 0.4) | ‑0.4 (‑1.3, 0.4) | 0.0 (‑1.0, 1.0) | 1.0000 |
| EPEN-18mg (n=39) | No Product (n=37) | ‑0.6 (‑1.4, 0.3) | ‑0.4 (‑1.3, 0.4) | ‑0.1 (‑1.1, 0.9) | 0.9980 |
| B734 (n=39) | No Product (n=37) | 0.1 (‑0.7, 0.9) | ‑0.4 (‑1.3, 0.4) | 0.5 (‑0.5, 1.5) | 0.6230 |
| EPEN-12mg (n=37) | EPEN-0mg (n=37) | ‑0.4 (‑1.2, 0.4) | ‑0.2 (‑1.1, 0.6) | ‑0.2 (‑1.2, 0.8) | 0.9838 |
| EPEN-18mg (n=39) | EPEN-0mg (n=37) | ‑0.6 (‑1.4, 0.3) | ‑0.2 (‑1.1, 0.6) | ‑0.3 (‑1.3, 0.7) | 0.8981 |
| B734 (n=39) | EPEN-0mg (n=37) | 0.1 (‑0.7, 0.9) | ‑0.2 (‑1.1, 0.6) | 0.3 (‑0.7, 1.3) | 0.9231 |
| EPEN-18mg (n=39) | EPEN-12mg (n=37) | ‑0.6 (‑1.4, 0.3) | ‑0.4 (‑1.2, 0.4) | ‑0.1 (‑1.1, 0.9) | 0.9967 |
| B734 (n=39) | EPEN-12mg (n=37) | 0.1 (‑0.7, 0.9) | ‑0.4 (‑1.2, 0.4) | 0.5 (‑0.5, 1.5) | 0.6564 |
| B734 (n=39) | EPEN-18mg (n=39) | 0.1 (‑0.7, 0.9) | ‑0.6 (‑1.4, 0.3) | 0.6 (‑0.4, 1.6) | 0.4086 |

Results obtained using an ANOVA on post‑product use results with fixed effects of product, period, sequence and a random effect of subject nested within sequence. Subjects with assessments flagged to indicate non‑compliance, who also meet the performance outlier threshold (+/‑ 1.5 x IQR), have been excluded from the analysis of this data

(ANOVA = analysis of variance, IQR = interquartile range). Baseline defined as ‑85 to ‑45 min pre‑product use.

- 1. *OTS Median Latency to First Choice (OTSMDLFC)*

| **Test Product** | **Reference Product** | **LSMean (95% CI)** | | **Difference in LSMeans (95% CI) Test ‑ Reference** | **Tukey Adjusted P‑value** |
| --- | --- | --- | --- | --- | --- |
|  |  | **Test Product** | **Reference Product** |  |  |
| EPEN-0mg (n=38) | No Product (n=37) | ‑1887.048 (‑2503.574, ‑1270.521) | ‑1548.037 (‑2167.822, ‑928.253) | ‑339.010 (‑1099.687, 421.667) | 0.7330 |
| EPEN-12mg (n=38) | No Product (n=37) | ‑1305.888 (‑1923.979, ‑687.798) | ‑1548.037 (‑2167.822, ‑928.253) | 242.149 (‑523.266, 1007.565) | 0.9059 |
| EPEN-18mg (n=39) | No Product (n=37) | ‑1344.376 (‑1957.908, ‑730.844) | ‑1548.037 (‑2167.822, ‑928.253) | 203.661 (‑559.181, 966.504) | 0.9473 |
| B734 (n=39) | No Product (n=37) | ‑1163.384 (‑1774.767, ‑552.002) | ‑1548.037 (‑2167.822, ‑928.253) | 384.653 (‑373.445, 1142.752) | 0.6274 |
| EPEN-12mg (n=38) | EPEN-0mg (n=38) | ‑1305.888 (‑1923.979, ‑687.798) | ‑1887.048 (‑2503.574, ‑1270.521) | 581.159 (‑174.797, 1337.116) | 0.2156 |
| EPEN-18mg (n=39) | EPEN-0mg (n=38) | ‑1344.376 (‑1957.908, ‑730.844) | ‑1887.048 (‑2503.574, ‑1270.521) | 542.672 (‑222.784, 1308.127) | 0.2916 |
| B734 (n=39) | EPEN-0mg (n=38) | ‑1163.384 (‑1774.767, ‑552.002) | ‑1887.048 (‑2503.574, ‑1270.521) | 723.663 (‑34.661, 1481.987) | 0.0691 |
| EPEN-18mg (n=39) | EPEN-12mg (n=38) | ‑1344.376 (‑1957.908, ‑730.844) | ‑1305.888 (‑1923.979, ‑687.798) | ‑38.488 (‑809.376, 732.400) | 0.9999 |
| B734 (n=39) | EPEN-12mg (n=38) | ‑1163.384 (‑1774.767, ‑552.002) | ‑1305.888 (‑1923.979, ‑687.798) | 142.504 (‑619.735, 904.743) | 0.9856 |
| B734 (n=39) | EPEN-18mg (n=39) | ‑1163.384 (‑1774.767, ‑552.002) | ‑1344.376 (‑1957.908, ‑730.844) | 180.992 (‑563.595, 925.579) | 0.9622 |

Results obtained using an ANOVA on post‑product use results with fixed effects of product, period, sequence and a random effect of subject nested within sequence. Subjects with assessments flagged to indicate non‑compliance, who also meet the performance outlier threshold (+/‑ 1.5 x IQR), have been excluded from the analysis of this data

(ANOVA = analysis of variance, IQR = interquartile range). Baseline defined as ‑85 to ‑45 min pre‑product use.

1. PAL
   1. *PAL Total Attempts Adjusted – PALTEA*

| **Test Product** | **Reference Product** | **LSMean (95% CI)** | | **Difference in LSMeans (95% CI) Test ‑ Reference** | **Tukey Adjusted P‑value** |
| --- | --- | --- | --- | --- | --- |
|  |  | **Test Product** | **Reference Product** |  |  |
| EPEN-0mg (n=38) | No Product (n=37) | ‑0.3 (‑2.4, 1.7) | 0.6 (‑1.5, 2.6) | ‑0.9 (‑4.2, 2.4) | 0.9403 |
| EPEN-12mg (n=39) | No Product (n=37) | ‑0.8 (‑2.8, 1.2) | 0.6 (‑1.5, 2.6) | ‑1.3 (‑4.6, 1.9) | 0.7812 |
| EPEN-18mg (n=39) | No Product (n=37) | ‑1.7 (‑3.7, 0.3) | 0.6 (‑1.5, 2.6) | ‑2.3 (‑5.5, 1.0) | 0.3026 |
| B734 (n=39) | No Product (n=37) | ‑0.4 (‑2.4, 1.6) | 0.6 (‑1.5, 2.6) | ‑1.0 (‑4.2, 2.2) | 0.9185 |
| EPEN-12mg (n=39) | EPEN-0mg (n=38) | ‑0.8 (‑2.8, 1.2) | ‑0.3 (‑2.4, 1.7) | ‑0.4 (‑3.7, 2.8) | 0.9958 |
| EPEN-18mg (n=39) | EPEN-0mg (n=38) | ‑1.7 (‑3.7, 0.3) | ‑0.3 (‑2.4, 1.7) | ‑1.4 (‑4.6, 1.9) | 0.7705 |
| B734 (n=39) | EPEN-0mg (n=38) | ‑0.4 (‑2.4, 1.6) | ‑0.3 (‑2.4, 1.7) | ‑0.1 (‑3.3, 3.1) | 1.0000 |
| EPEN-18mg (n=39) | EPEN-12mg (n=39) | ‑1.7 (‑3.7, 0.3) | ‑0.8 (‑2.8, 1.2) | ‑0.9 (‑4.1, 2.3) | 0.9308 |
| B734 (n=39) | EPEN-12mg (n=39) | ‑0.4 (‑2.4, 1.6) | ‑0.8 (‑2.8, 1.2) | 0.4 (‑2.8, 3.5) | 0.9979 |
| B734 (n=39) | EPEN-18mg (n=39) | ‑0.4 (‑2.4, 1.6) | ‑1.7 (‑3.7, 0.3) | 1.3 (‑1.9, 4.4) | 0.7963 |

Results obtained using an ANOVA on post‑product use results with fixed effects of product, period, sequence and a random effect of subject nested within sequence. Subjects with assessments flagged to indicate non‑compliance, who also meet the performance outlier threshold (+/‑ 1.5 x IQR), have been excluded from the analysis of this data

(ANOVA = analysis of variance, IQR = interquartile range).

Baseline defined as ‑85 to ‑45 min pre‑product use

- 1. *PAL First Attempt Memory Score (PALFAMS)*

| **Test Product** | **Reference Product** | **LSMean (95% CI)** | | **Difference in LSMeans (95% CI) Test ‑ Reference** | **Tukey Adjusted P‑value** |
| --- | --- | --- | --- | --- | --- |
|  |  | **Test Product** | **Reference Product** |  |  |
| EPEN-0mg (n=38) | No Product (n=37) | ‑0.1 (‑1.0, 0.8) | ‑0.3 (‑1.2, 0.6) | 0.2 (‑1.4, 1.8) | 0.9947 |
| EPEN-12mg (n=39) | No Product (n=37) | 0.2 (‑0.7, 1.1) | ‑0.3 (‑1.2, 0.6) | 0.5 (‑1.1, 2.1) | 0.9257 |
| EPEN-18mg (n=39) | No Product (n=37) | 0.4 (‑0.5, 1.3) | ‑0.3 (‑1.2, 0.6) | 0.7 (‑0.9, 2.3) | 0.7499 |
| B734 (n=39) | No Product (n=37) | 0.3 (‑0.6, 1.2) | ‑0.3 (‑1.2, 0.6) | 0.6 (‑1.0, 2.2) | 0.8523 |
| EPEN-12mg (n=39) | EPEN-0mg (n=38) | 0.2 (‑0.7, 1.1) | ‑0.1 (‑1.0, 0.8) | 0.2 (‑1.4, 1.8) | 0.9936 |
| EPEN-18mg (n=39) | EPEN-0mg (n=38) | 0.4 (‑0.5, 1.3) | ‑0.1 (‑1.0, 0.8) | 0.5 (‑1.1, 2.1) | 0.9283 |
| B734 (n=39) | EPEN-0mg (n=38) | 0.3 (‑0.6, 1.2) | ‑0.1 (‑1.0, 0.8) | 0.4 (‑1.2, 1.9) | 0.9733 |
| EPEN-18mg (n=39) | EPEN-12mg (n=39) | 0.4 (‑0.5, 1.3) | 0.2 (‑0.7, 1.1) | 0.2 (‑1.4, 1.8) | 0.9950 |
| B734 (n=39) | EPEN-12mg (n=39) | 0.3 (‑0.6, 1.2) | 0.2 (‑0.7, 1.1) | 0.1 (‑1.5, 1.7) | 0.9997 |
| B734 (n=39) | EPEN-18mg (n=39) | 0.3 (‑0.6, 1.2) | 0.4 (‑0.5, 1.3) | ‑0.1 (‑1.7, 1.5) | 0.9996 |

Results obtained using an ANOVA on post‑product use results with fixed effects of product, period, sequence and a random effect of subject nested within sequence. Subjects with assessments flagged to indicate non‑compliance, who also meet the performance outlier threshold (+/‑ 1.5 x IQR), have been excluded from the analysis of this data

(ANOVA = analysis of variance, IQR = interquartile range). Baseline defined as ‑85 to ‑45 min pre‑product use.

***File S3***

*Summary of the Statistical Analysis of Questionnaire on Smoking Urges – Brief (QSU-Brief)*

|  | **Test Product** | **Reference Product** | **LSMean (95% CI)** | | **Difference in LSMeans**  **(95% CI) Test ‑ Reference** | **Tukey Adjusted P‑value** |
| --- | --- | --- | --- | --- | --- | --- |
|  |  |  | **Test Product** | **Reference Product** |  |  |
| **Factor 1**  **(Positive Reinforcement Properties of Smoking)** | EPEN-0mg (n=38) | No Product  (n=37) | ‑1.6  (‑3.6, 0.4) | 1.4  (‑0.7, 3.4) | ‑2.9  (‑6.2, 0.4) | 0.1186 |
|  | EPEN-12mg (n=39) | No Product  (n=37) | ‑4.8  (‑6.7, ‑2.8) | 1.4  (‑0.7, 3.4) | ‑6.1  (‑9.4, ‑2.8) | <.0001 |
|  | EPEN-18mg (n=39) | No Product  (n=37) | ‑7.2  (‑9.2, ‑5.2) | 1.4  (‑0.7, 3.4) | ‑8.5  (‑11.9, ‑5.2) | <.0001 |
|  | B734 (n=39) | No Product  (n=37) | ‑10.7  (‑12.6, ‑8.7) | 1.4  (‑0.7, 3.4) | ‑12.0  (‑15.3, ‑8.7) | <.0001 |
|  | EPEN-12mg (n=39) | EPEN-0mg (n=38) | ‑4.8  (‑6.7, ‑2.8) | ‑1.6  (‑3.6, 0.4) | ‑3.2  (‑6.5, 0.1) | 0.0602 |
|  | EPEN-18mg (n=39) | EPEN-0mg (n=38) | ‑7.2  (‑9.2, ‑5.2) | ‑1.6  (‑3.6, 0.4) | ‑5.6  (‑8.9, ‑2.3) | <.0001 |
|  | B734 (n=39) | EPEN-0mg (n=38) | ‑10.7  (‑12.6, ‑8.7) | ‑1.6  (‑3.6, 0.4) | ‑9.1  (‑12.4, ‑5.8) | <.0001 |
|  | EPEN-18mg (n=39) | EPEN-12mg (n=39) | ‑7.2  (‑9.2, ‑5.2) | ‑4.8  (‑6.7, ‑2.8) | ‑2.4  (‑5.7, 0.8) | 0.2469 |
|  | B734 (n=39) | EPEN-12mg (n=39) | ‑10.7  (‑12.6, ‑8.7) | ‑4.8  (‑6.7, ‑2.8) | ‑5.9  (‑9.2, ‑2.6) | <.0001 |
|  | B734 (n=39) | EPEN-18mg (n=39) | ‑10.7  (‑12.6, ‑8.7) | ‑7.2  (‑9.2, ‑5.2) | ‑3.5  (‑6.7, ‑0.2) | 0.0294 |
| **Factor 2**  **(Negative Reinforcement Properties of Smoking)** | EPEN-0mg (n=38) | No Product  (n=37) | ‑0.9  (‑2.1, 0.3) | 1.1  (‑0.1, 2.3) | ‑2.0  (‑3.9, 0.0) | 0.0568 |
|  | EPEN-12mg (n=39) | No Product  (n=37) | ‑2.5  (‑3.6, ‑1.3) | 1.1  (‑0.1, 2.3) | ‑3.6  (‑5.5, ‑1.6) | <.0001 |
|  | EPEN-18mg (n=39) | No Product  (n=37) | ‑3.7  (‑4.9, ‑2.5) | 1.1  (‑0.1, 2.3) | ‑4.8  (‑6.8, ‑2.8) | <.0001 |
|  | B734 (n=39) | No Product  (n=37) | ‑4.8  (‑5.9, ‑3.6) | 1.1  (‑0.1, 2.3) | ‑5.8 (  ‑7.8, ‑3.9) | <.0001 |
|  | EPEN-12mg (n=39) | EPEN-0mg (n=38) | ‑2.5  (‑3.6, ‑1.3) | ‑0.9  (‑2.1, 0.3) | ‑1.6  (‑3.6, 0.4) | 0.1671 |
|  | EPEN-18mg (n=39) | EPEN-0mg (n=38) | ‑3.7  (‑4.9, ‑2.5) | ‑0.9  (‑2.1, 0.3) | ‑2.8  (‑4.8, ‑0.9) | 0.0011 |
|  | B734 (n=39) | EPEN-0mg (n=38) | ‑4.8  (‑5.9, ‑3.6) | ‑0.9  (‑2.1, 0.3) | ‑3.9  (‑5.8, ‑1.9) | <.0001 |
|  | EPEN-18mg (n=39) | EPEN-12mg (n=39) | ‑3.7  (‑4.9, ‑2.5) | ‑2.5  (‑3.6, ‑1.3) | ‑1.2  (‑3.2, 0.7) | 0.4126 |
|  | B734 (n=39) | EPEN-12mg (n=39) | ‑4.8  (‑5.9, ‑3.6) | ‑2.5  (‑3.6, ‑1.3) | ‑2.3  (‑4.2, ‑0.3) | 0.0133 |
|  | B734 (n=39) | EPEN-18mg (n=39) | ‑4.8  (‑5.9, ‑3.6) | ‑3.7  (‑4.9, ‑2.5) | ‑1.1  (‑3.0, 0.9) | 0.5706 |

Factor 1 = sum of Q1, Q3, Q6, Q7 and Q10 scores. Factor 2 = sum of Q4, Q8 and Q9 scores. Baseline defined as pre‑IP use.

Results obtained using an ANOVA with fixed effects of product, period, sequence and a random effect of subject nested within sequence. (ANOVA = analysis of variance, IP = investigational product)
